# Implementing cognitive remediation and social cognitive interaction training into standard psychosis care

**DOI:** 10.1186/s12913-018-3240-5

**Published:** 2018-06-15

**Authors:** Frances Dark, Meredith Harris, Victoria Gore-Jones, Ellie Newman, Harvey Whiteford

**Affiliations:** 1 Rehabilitation Academic Clinical Unit, Metro South Addiction and Mental Health Service, 228 Logan Rd, Woolloongabba, QLD Australia; 2 School of Medicine, The University of Queensland, Herston, QLD Australia; 3 School of Public Health, The University of Queensland, Herston, QLD Australia; 4 Policy and Epidemiology Group, Queensland Centre for Mental Health Research, Wacol, QLD Australia; 5 Metro South Addiction and Mental Health Service, 228 Logan Rd, Woolloongabba, QLD Australia

**Keywords:** Implementation outcomes, Cognitive remediation, Social cognitive interaction training, Schizophrenia, Mental health services

## Abstract

**Background:**

To evaluate the planned implementation of group based Cognitive Remediation therapy (CR) and Social Cognitive Interaction Training (SCIT) into routine psychosis care in a mental health service in Australia.

**Method:**

The study was conducted over 3 years in a mental health service in a metropolitan city in Australia. Participants were 22 program facilitators and 128 patients attending the programs. Implementation outcomes were assessed using administrative data, staff surveys and program audits.

**Results:**

There was fidelity to the particular therapies at a program level. Programs were assessed as being feasible within the study setting with each hospital district developing a capacity to run CR and SCIT. The establishment of new programs improved the reach, but waiting lists indicate a need to expand capacity. There was a relatively high dropout and several factors impacted on completion of the programs - notably, acute exacerbation of psychosis. Once initiated the therapies were acceptable with no-one ceasing SCIT due to loss of interest and only 10% of participants ceasing CR due to loss of interest. Annual audits of programs found programs established were maintained and facilitators were retained.

**Conclusion:**

SCIT and CR programs were successfully implemented in three hospital districts. Several factors impeded participants receiving the recommended “dose” of the programs. The maintenance of the programs in the short term is encouraging in regards to organisational fit. Dissemination of cognitive rehabilitation programs to a service population takes planning. An implementation plan is essential for guiding development and maintenance of programs. These therapies are best suited to people in a stable phase of illness. Service user co-production is recommended to improve recruitment in future studies.

**Electronic supplementary material:**

The online version of this article (10.1186/s12913-018-3240-5) contains supplementary material, which is available to authorized users.

## Background

Comprehensive care for people diagnosed with psychosis includes access to adjunctive evidence based psychosocial interventions [1, 2]. Cognitive remediation therapies for psychosis are recommended in the recently updated Australian and New Zealand College of Psychiatrists clinical practice guidelines [1]. Translating clinical guideline recommendations for psychosocial interventions into routine practice has been a challenge [3, 4]. Implementation science provides a framework to guide the translation of evidence based therapies into routine care [5]. This approach champions external validity and engagement with real world clinical settings and practice.

In an implementation framework program implementation outcomes (fidelity, feasibility, reach, acceptability, maintenance, and costs) are delineated as a distinct outcome category that influences service and clinical program outcomes [6]. Improved clinical outcomes are seen as arising from the marriage of interventions with robust evidence with effective implementation strategies [7]. Fidelity refers to the delivery of the intervention as proven to be effective and can be measured at an individual therapist level or at a program level. Feasibility assesses if the intervention has utility for routine use in usual care settings and can be assessed at an individual therapist/ participant level and/or an organisational level. Reach is the level of dissemination within an organisation and is related to treatment access and can be assessed via auditing programs within a population. Acceptability relates to satisfaction with the program and can be assessed at an individual provider or participant level. Maintenance of programs refers to the ongoing use of a program after the initial implementation and depends on the degree to which the program becomes embedded within the usual business of the service and can be assessed via regular audits. Cost of implementing new programs relates to new labour and non-labour establishment and recurrent costs and is of particular concern to the financial management of the provider organisation.

Cognitive impairment is common in people diagnosed with schizophrenia [8, 9]. The deficits are global and impact on both neuro cognition (attention, memory, planning) and social cognition (difficulties perceiving and processing emotions) [9, 10]. Approaches to remediation of these deficits have included integrated programs with a combined approach to neuro cognition and social cognition and also stand-alone programs focussing either on neuro cognition or social cognition [11, 12]. There is evidence from a meta-analysis of randomized controlled trials for the benefit of Cognitive Remediation therapy (CR) on global cognition (Effect Size ES =0.45) with greater benefit if CR is combined with some form of rehabilitation (ES = 0.59) [11]. The core components of CR appear to be: intensity of practice (2–4 times a week); the combination of drill and practice of tasks with strategy training and a context that facilitates use of new skills learnt. In the meta-analysis, 14 different CR programs were examined with no particular program having superior effect [11].

There is also evidence from a meta-analysis of the effectiveness of interventions remediating social cognitive deficits in schizophrenia [12]. Social Cognitive Interaction Training (SCIT) is one example of a social cognition remediation program. It is comprised of three phases (Introduction & Emotions, Figuring out Situations, Checking it out) over 20 to 24 sessions. It is administered in a group format [13]. It has been delivered in weekly hour-long sessions (20–24 weeks), and weekly 2-h sessions (10-12 weeks) [14].

Four CR programs and four SCIT programs had been established in Brisbane’s Princess Alexandra Hospital (PAH), and the associated health district prior to this study commencing. These programs commenced following staff training in CR in 2008 and SCIT in 2010. In 2012 the service restructured into teams based on diagnosis or model of service (e.g. rehabilitation) to encourage the development of core diagnostically relevant evidence based practices and to more equitably distribute resources. This restructure gave organisational impetus to apply implementation theory to the issue of limited implementation and dissemination of cognitive therapies for psychosis.

Staff from the service involved in psychosis care, Psychosis Academic Clinical Unit (ACU) and Rehabilitation ACU in which the study was carried out developed a plan to guide implementation of CR and SCIT in the 3 geographically defined hospital health districts of the service. The implementation plan was organised around the stages of implementation: exploration/adoption; initial implementation; full implementation and program maintenance [7]. The exploration/adoption stage had been completed with pilot studies in one hospital site [15, 16]. The baseline organisational factors were assessed [17]. The initial implementation focussed on the implementation drivers with the appointment of implementation champions, the formation of the implementation team, the securing of infrastructure for each sites and the establishment of referral pathways and regular training and supervision of staff. The full implementation stage involved annual program audits, and annual program fidelity monitoring. The CR program fidelity for programs implemented in the first year of the study has previously been reported [18].The maintenance stage involved securing ongoing program funding support, regular training and the development of a pool of program specialists who were involved in providing supervision and iterative program improvements. This study reports on the implementation outcomes of program fidelity, feasibility, reach, acceptability, maintenance and cost, three years into the implementation process.

## Method

### Design and setting

This evaluation study was conducted over three years from 2013 to 2015 and collected information on the implementation outcomes (fidelity, feasibility, reach, acceptability, maintenance, and costs) of CR and SCIT programs implemented.

The study setting was the community mental health services of a metropolitan health service (MHS) with a population of around 920,000 in Brisbane, Australia. The health service comprises three hospital health districts (PAH, Logan and Bayside). It has been estimated that approximately three thousand people residing in this area have severe and persistent mental illness with complex needs (Greater Metro South Brisbane Medicare Local Schedule 10, 2013). The area has high rates of socioeconomic disadvantage with 19.8% of people postponing mental health care because of cost (Greater Metro South Brisbane Medicare Local Schedule 10, 2013). The geographic area covers inner metropolitan suburbs to semi-rural areas. There are three hospitals in the geographically defined health districts.

### Population

#### Program facilitators

Both programs were facilitated by a multidisciplinary group of therapists who had identified an interest in becoming CR and SCIT therapists and had completed the requisite training and credentialing [18]. The therapy was undertaken as part of their general role in the service with therapy time quarantined and case weighting altered to facilitate staff capacity to undertake this work. There were 12 cognitive remediation therapists (4 psychologists, 2 occupational therapists, 2 psychiatrists, 3 mental health nurses and 1 social worker). There were 10 SCIT therapists (5 psychologists, 2 psychiatrist, 2 nurses and 1 social worker).

#### Program participants

People diagnosed with a schizophrenia spectrum disorder who were being treated in the community, aged between 18 and 65, and who did not have an intellectual impairment or active substance abuse disorder were eligible to participate. Service users could participate in the program and not consent to the evaluation research project.

#### Cognitive rehabilitation therapies implemented

The characteristics of the programs implemented are listed in Table 1. The specific CR interventions used in this study were a computer based program, CIRCuiTS (Computerized Interactive Remediation of Cognitive and Thinking Skills) [19] and a program based on educational software using computers, Neuro psychological Educational Approach to Cognitive Remediation (NEAR) program [20]. Both programs run twice a week for around an hour. CR is run as an open group with 4 participants to one therapist.

**Table 1:** CR and SCIT program components

	CR	SCIT
Components	• Computer program • 15 min bridging group	• Manualised therapy
Frequency	• Twice a week	• Once a week
Duration	• 10–20 weeks	• 10–12 weeks
Intensity	• 60 min	• 120 min
Mode of delivery	• Computer session 45 min + 15 min bridging small group • Open group	• Small group = 8 • 2 therapists • Closed group
Materials	• Computer • Bridging manual	• Facilitators Manual • Participants Manual

SCIT is based on a manual and uses some resources available freely online. SCIT is run as a closed small group of a maximum of 8 participants and 2 facilitators. The program is structured to build skills over 3 phases focussing in phase 1 on emotional processing; phase 2 focussing on thinking skills in social situations; and phase 3 integrating these skills into real life social situations experienced by participants.

### Measures

#### Data sources

Implementation outcomes were assessed using data from multiple sources including yearly staff surveys, program audits, and measures of program feasibility.

#### Yearly staff surveys (Additional file 1)

This survey asked questions about staff interest, training and current practice with 8 questions about cognitive behavioural therapy (with SCIT classified as a CBT derived therapy) and 5 questions related to CR. The survey was distributed yearly for the three years of the study to clinical staff in the community psychosis and rehabilitation teams.

#### Annual program audits

Program audits were conducted annually by the program co-ordinator VGJ. For CR the number of programs running in each service, the maintenance of programs established, the number and retention of facilitators, the number of computers and funding was recorded. For SCIT the number of programs conducted and number and retention facilitators was noted.

#### Annual fidelity checks

No fidelity instrument had been developed for CIRCuiTS. Program fidelity was therefore measured using an instrument developed by Alice Medalia and colleagues for the Neuro psychological Educational Approach to Cognitive Remediation (NEAR) program which was adapted for the CIRCuiTS and SCIT programs (Medalia and Freilich, 2008). There were 12 criteria for CR programs: clinician credentials and training; supervision; software used and selection (NEAR only); referral process; assessment; personalised folders; application of assessment; specific CR techniques used; intrinsic motivation; time on each program (NEAR only); software features (NEAR only) and communication with referring team. The 7 criteria for SCIT program fidelity were: clinician credentials and training; supervision; manual adherence; referral process; assessment process; application of assessment; and demonstration of specific SCIT techniques. Each criterion was rated by FD on a 5 point scale: from not achieved; occasionally achieved; frequently achieved; infrequently not achieved; and 100% adherence.

#### Acceptability measures

A three-item measure of transportability, acceptability and feasibility was used by the SCIT program facilitators at the end of the program. This measure had been used in a previous study by the SCIT program developers [21]. The items asked if program participation was useful, if it helped thinking in social situations and helped to relate to others. Participants and referring clinical staff were asked to rate their responses on a 7 point (1 not helpful, 4 unsure and 7 very helpful) Likert scale. This measure was administered post-treatment.

This measure has not been previously used in CR studies and was not used to evaluate the feasibility or acceptability of CR in this study. The number of people referred to CR who commenced, whether consenting to the research or not, and reasons for discontinuing the program was used as a proxy measure of acceptability of the program.

### Program costs

Cost outlays for the programs included labour and non-labour expenses over the three years. Labour costs were calculated based on an average therapist’s hourly award wage. Therapists spent 2 h per week conducting the groups with SCIT being held once a week for 2 h and CR 1-h sessions twice a week. The costs of acquiring new non-labour items were included in the calculation. For CR this included the cost of purchasing computers and internet connection.

### Data analysis

Descriptive statistics were used to report the implementation outcomes.

## Results

### Program population

The consenting participants who commenced CR (*n* = 40) were aged between 26 and 60 with a mean age of 42 (SD = 10.4). They had between 6 and 15 years of education with a mean of 10.4 years (SD =2.0). More males (67%) than females (33%) consented to the study. Twenty-five percent were living in Department of Housing accommodation; 25% were living in private dwellings; 40% in residential rehabilitation units; and 10% where the accommodation was unspecified. Of the 19 participants who completed 10 or more sessions, 68% were male and 32% female.

The age range of participants consenting and who commenced SCIT (*n* = 33) was 19 to 50 years with a mean of 30.9 years (SD = 8.6 years). They had between 6 and 15 years of education with a mean of 10.8 years (SD = 2.2 years). More males (67%) than females (33%) consented to the study. Fifty- 1 % lived in a residential rehabilitation unit; 46% in a private dwelling; and 3% accommodation was unspecified. Of the 22 participants who completed 16 or more sessions, 64% were male and 36% female.

### Fidelity

All CR program facilitators had completed the two day training and were observed to be competent by an experienced facilitator. A clear referral and assessment process was followed by all staff. All programs were rated as having frequent fidelity to the specific CR techniques with no ratings on any occasion falling below frequently achieved. Each program had different processes for feedback to treating teams with no standard process. Monthly centralised supervision was accessed routinely by facilitators from only 1 program.

All staff facilitating SCIT were trained and experienced facilitators were coupled with less experienced facilitators. Fidelity to the treatment manual was discussed and reviewed by the facilitators following each session to minimize “therapeutic drift”. All programs were rated as having frequent fidelity to the specific SCIT techniques with no ratings on any occasion falling below “frequently achieved”. Each program had different processes for feedback to treating teams (e.g. informal verbal feedback to case managers, regular feedback in team meetings).

### Feasibility

All 3 hospitals in the geographically defined health districts participated. The estimated total population of patients diagnosed with schizophrenia spectrum disorders in the service at the time of the study was 1660. From this population 105 people were referred to the CR programs and 53 were referred for the SCIT programs. All sites had staff from multiple disciplines trained and competent to deliver both therapies. CR was run as an open group. The SCIT groups were run as a closed group once or twice a year. Three SCIT programs were conducted in the hospital district that had previously run pilot SCIT programs and 1 program ran at each of the other two hospital districts.

### Reach

Each hospital district was serviced by at least 1 CR and 1 SCIT program. The inner city hospital district had 4 CR programs established and 3 SCIT programs were conducted. CR was conducted at the residential rehabilitation units but accepted referrals from the community clinics in Logan and Bayside Hospitals and districts. The referring community clinics have made requests for access to programs that were at full capacity necessitating the creation of waiting lists. Based on this unmet demand a further 2 CR programs and 2 SCIT programs are estimated to be needed for the community teams.

### Acceptability

Of the 89 people referred to CR, 52 consented to participating in the evaluation research. Twelve of the 52 consenting failed to commence the program. Of the 40 participants consenting and commencing the CR program, 21 completed at least 10 sessions. Nineteen of the research participants discontinued CR before completing 10 sessions. Seven participants had time constraints, usually due to family responsibilities; 3 were hospitalised due to acute deterioration in mental state; 4 moved area and had trouble accessing the program and for one participant no reason for discontinuation was given. All cases of acute deterioration in mental health were considered by their clinical team as not relating to the programs. Where possible an interview of participants on exiting the program early was used to explore satisfaction with the CR program. Only 4 participants (10%) stopped attending CR due to loss of interest in the program.

Of the 53 eligible people referred to SCIT, 39 consented to be involved in the study. Six of the 39 did not commence. Of the 33 commencing SCIT, 24 completed more than 16 sessions which was considered as having completed the program. Eight failed to complete the program due to acute deterioration requiring hospitalisation and no reason was recorded in one case. All cases of acute deterioration in mental health were considered by their clinical team as not relating to the programs. Program acceptability was recorded for SCIT using a three item measure (Table 2). On average, based on a scale of 1 to 7 both participants and staff considered the program useful in relation to social thinking and relating. The participants’ average scores were above 5 with a range of 4 to 7 for “program participation was useful” and “program participation helped in thinking in social situations” criteria and 3–7 for “program participation helped me to relate to others”.

**Table 2:** Participant and therapist acceptability and feasibility ratings SCIT

	Participant Mean Range	Clinicians Mean Range
Participation in the group was useful to me/my client	5.1 (4–7)	5.2 (3–7)
The group helped me/ my client think about social situations	5.6 (4–7)	5.3 (2–7)
The group helped me/ my client to relate to other people	5.2 (3–7)	5.4 (4–7)

### Maintenance

Over the 3 years of the study (2013–2015) the number of CR programs doubled to 8. The 3 programs running in the PAH district and 1 program running at Logan hospital district at baseline were still active at the end of the study period. In 2014 an additional 2 programs were established (1 Logan, and 1 Bayside). Another 2 programs were established in Bayside in 2015. All established programs were maintained at the last audit in December 2015. In 2013 there were 10 program facilitators. These facilitators were retained and 3 new trained facilitators commenced in 2014. The 13 trained facilitators were retained at the end point audit in December 2015. In 2014 ongoing funding was secured to maintain internet connection and 12 new computers were purchased to replace old computers and to support the new programs.

SCIT programs were run each six months in the PAH hospital district (2013–2015) and one program at Bayside and Logan in the last year of the project. The PAH had previous experience with SCIT and the Bayside and Logan programs were run at the residential rehabilitation services that had only opened in 2015. These programs are ongoing following the end of the study and scheduled to run every six months depending on demand. Ten therapists were trained in the mental health service and were still running groups at the last audit in 2015.

### Costs

The total expenditure on CR programs including labour and non-labour was $32,848 over the three years (2013–2015). The use of some donated computers lowered costs for purchasing computers. The SCIT programs involved only labour costs of $11,520 (Table 3).

**Table 3:** Total program cost outlays and cost offsets over three years (2013–2015)

	CR Total costs	SCIT Total costs
Computers	$4000	N/R
Internet	$1200	N/R
Staff × 2 (estimated therapy hours x rate of pay) $48/h	$27,648/3 years	$11,520/3 years
Expenditure	$32,848	$11,520

## Discussion

Studies of the implementation of cognitive rehabilitation therapies into routine mental health care using implementation theory are beginning to emerge [17, 18, 22–24]. The implementation plan described in this paper resulted in CR and SCIT programs being disseminated throughout a large health service comprising three hospital districts. There was organisational executive support for the development of evidence based programs with the service reorganisation in 2012. Organisational support has been found to be necessary but not sufficient for successful program implementation [7].

The organisational support and previous exploration of the cognitive rehabilitation therapies of interest, resulted in a plan to systematically implement CR and SCIT. CR and SCIT had been run in one hospital district since 2008 and 2010 respectively with no programs existing in two other hospital districts [15, 16]. This is consistent with the implementation literature that has found passive diffusion of innovations are not as successful as planned and staged implementation [7]. The growth in the number of programs over the years of the study is also consistent with the 2 to 4 year project plan required for most implementation projects [7].

The current evaluation study found the programs were feasible within services more distal to the largest hospital in the area. External factors to an organisation may determine the feasibility of decentralized or centralising programs. Centralized well-resourced program “hubs” may be acceptable and accessible if transport is convenient and affordable. Program maintenance and quality can be a challenge with dissemination to smaller, less resourced centres. In this study the programs were disseminated out from the largest hospital (PAH) that had established the initial programs to the other two hospitals (Logan and Bayside) due to the large geographical area and the relative expense of public transport for this population. Support for these programs was provided from the central “hub” via supervision either face to face or via teleconferencing. Four participants who moved areas failed to complete CR implying that lack of local access can influence the feasibility of attending programs.

For new programs to become embedded in routine care, the organisational fit, feasibility and acceptability of the programs to staff need to be considered [25]. In this study, findings regarding maintenance of programs and retention of facilitators were interpreted as proxy measures indicating these programs demonstrated an acceptable organisational fit.

Reasons for discontinuing CR early were collected. The overall attrition rate for those consenting to the evaluation was high (55%). Wykes in a meta-analysis found the overall attrition rate for CR to be 11% with a range of 0–47.5% (Wykes et al., 2011). In this study in relation to acceptability, once initiated only 10% ceased the CR program because of loss of interest. In both programs acute illness resulted in early exit from the programs reflecting the relapsing nature of schizophrenia. Accurate assessment of the utilization of the CR program was not possible due to the low rates of participants consenting to research (i.e., 37, 42%attended CR but did not consent to the evaluation). Previous pilot studies of SCIT found lower attrition in participants living in residential settings [15]. A significant number of participants were recruited for SCIT from residential settings (40% CR and 51% SCIT participants). In this study 65% of non-completers of SCIT were living in the community. The support from staff in the residential settings to remind and to help motivate participants to attend may contribute to these lower attrition rates. This also supports current efforts to increase service user involvement in design and production of research, which has been shown to improve recruitment [26]. In the maintenance phase the programs need to become embedded into clinical governance procedures where utilization can be monitored as part of routine quality assurance practices.

A formal economic analysis was not included in the protocol. Cost outlays are higher for CR than for SCIT due to more sessions being conducted and the use of computers and internet (Table 3). Previous studies suggest that cognitive remediation programs may result in service cost savings [27].

### Limitations

Implementation studies are inherently context specific making extrapolation to other services difficult. These results are particularly relevant to the Australian public mental health sector. The higher program participation, while not consenting to research, also biases the results, limiting extrapolation to the population of people who attended the programs.

The 3 year implementation timeframe for this study is brief given the complexity of the interventions. The study site had experience in the programs (exploration phase of implementation) and this needs to be considered in relation to the overall time required to implement these programs.

The study did not involve a formal cost-effectiveness analysis. This information is needed to help prosecute the case for funding the implementation of these therapies in the context of increasingly constrained health budgets.

## Conclusions

Implementation theory and practice can help guide the implementation of evidence based therapies recommended into routine, comprehensive psychosis care. Having a staged plan over time is required to address the complex workforce, organisational and quality issues that are inherent in delivering cognitive therapies for psychosis. Service user involvement in the design and running of psychosocial programs is recommended.

## Additional file

Additional file 1: SSPARS BMC Staff survey. (DOC 28 kb)

## Abbreviations

ACU: Academic Clinical Unit
CBT: Cognitive Behaviour Therapy
CIRCuiTS: Computerised Interactive Remediation of Cognitive Thinking Skills
CR: Cognitive Remediation Therapy
ES: Effect Size
MHS: Metropolitan Health Service
NEAR: Neuropsychological Educational Approach to Cognitive Remediation
PAH: Princess Alexandra Hospital
SCIT: Social Cognitive Interaction Training

## References

[1] Galletly C, Castle D, Dark F, Humberstone V, Jablensky A, Killackey E, Kulkarni J, McGorry P, Nielssen O, Tran N (2016). Royal Australian and New Zealand College of Psychiatrists clinical practice guidelines for the management of schizophrenia and related disorders. Aust N Z J Psychiatry.

[2] Kuipers E, Yesufu-Udechuku A, Taylor C, Kendall T (2014). Management of psychosis and schizophrenia in adults: summary of updated NICE guidance. BMJ.

[3] Barbui C, Girlanda F, Ay E, Cipriani A, Becker T, Koesters M (2014). Implementation of treatment guidelines for specialist mental health care. Schizophr Bull.

[4] Michie S, Berentson-Shaw J, Pilling S, Feder G, Dieppe P, Raine R, Cluzeau F, Alderson P, Ellis S (2007). Turning evidence into recommendations: protocol of a study guideline development groups. Implement Sci.

[5] Corrigan PW, Steiner L, McCracken SG, Blaser B, Barr M (2001). Strategies for disseminating evidence based practices to staff who treat people with serious mental illness. Psychiatr Serv.

[6] Proctor EK (2009). Implementation research in mental health services: an emerging science with conceptual, methodological, and training challenges. J Adm Policy Mental Health.

[7] Fixsen D, Naoom S, Blase K, Friedman R, Wallace F (2005). Implementation research: a synthesis of the literature.

[8] Keefe RS, Eesley CE, Poe MP (2005). Defining a cognitive function decrement in schizophrenia. Biol Psychiatry.

[9] Bowie CR, Harvey PD (2005). Cognition in schizophrenia: impairments, determinants, and functional importance. Psychiatr Clin North Am.

[10] Fioravanti M, Bianchi V, Cinti ME (2012). Cognitive deficits in schizophrenia: an updated metanalysis of the scientific evidence. BMC Psychiatry.

[11] Wykes T, Huddy V, Cellard C, McGurk SR, Czobor P (2011). A meta-analysis of cognitive remediation for schizophrenia: methodology and effect sizes. Am J Psychiatr.

[12] Kurtz MM, Richardson CL (2012). Social cognitive training for schizophrenia: a meta-analytic investigation of controlled research. Schizophr Bull.

[13] Combs DR, Adams SD, Penn DL, Roberts D, Tiegreen J, Stem P (2007). Social cognition and interaction training (SCIT) for inpatients with schizophrenia spectrum disorders: preliminary findings. Schizophr Res.

[14] Bartholomeusz CF, Allott K, Killackey E, Liu P, Wood SJ, Thompson A (2013). Social cognition training as an intervention for improving functional outcome in first-episode psychosis: a feasibility study. Early Interv Psychiatry.

[15] Parker S, Foley S, Walker P, Dark F (2013). Improving the social cognitive deficits of schizophrenia: a community trial of social cognition and interaction training (SCIT). Australas Psychiatry.

[16] Cairns A, Dark F, Batts M (2013). Implementing cognitive remediation therapy: lessons from two public mental health services. Australas Psychiatry.

[17] Dark F, Whiteford H, Ashkanasy NM, Harvey C, Crompton D, Newman E (2015). Implementing cognitive therapies into routine psychosis care: organisational foundations. BMC Health Serv Res.

[18] Dark F, Newman E, Harris M, Cairns A, Simpson M, Gore-Jones V, Whiteford H, Harvey C, Crompton D (2016). Implementing cognitive remediation therapy (CRT) in a mental health service: staff training. Australas Psychiatry.

[19] Reeder C, Huddy V, Cella M, Taylor R, Greenwood K, Landau S, Wykes T (2017). A new generation computerised metacognitive cognitive remediation programme for schizophrenia (CIRCuiTS): a randomised controlled trial. Psychol Med.

[20] Medalia A, Freilich B (2008). The Neuropsychological Educational Approach to Cognitive Remediation (NEAR) Model: Practice Principles and Outcome Studies, vol. 11.

[21] Roberts DL, Penn DL, Labate D, Margolis SA, Sterne A (2009). Transportability and feasibility of social cognition and interaction training (SCIT) in community settings. Behav Cogn Psychother.

[22] Amado I, Sederer LI (2016). Implementing cognitive remediation programs in France: the “secret sauce”. Psychiatr Serv (Washington, DC).

[23] McGurk SR, Mueser KT, Watkins MA, Dalton CM, Deutsch H (2017). The feasibility of implementing cognitive remediation for work in community based psychiatric rehabilitation programs. Psychiatr Rehabil J.

[24] John AP, Yeak K, Ayres H, Dragovic M (2017). Successful implementation of a cognitive remediation program in everyday clinical practice for individuals living with schizophrenia. Psychiatr Rehabil J.

[25] Damschroder L, Aron D, Keith R, Krish S, Alexander J, Lowery J (2009). Fostering implementation of health service research findings into practice: a consolidation framework for advancing implementation science. Implement Sci.

[26] Ennis L, Wykes T (2013). Impact of patient involvement in mental health research: longitudinal study. Br J Psychiatry.

[27] Reeder C, Harris V, Pickles A, Patel A, Cella M, Wykes T (2014). Does change in cognitive function predict change in costs of care for people with a schizophrenia diagnosis following cognitive remediation therapy?. Schizophr Bull.

