# Supplementary material for: Implementing cognitive remediation and social cognitive interaction training into standard psychosis care
Source: BMC Health Serv Res. 2018 Jun 15;18:458. doi: 10.1186/s12913-018-3240-5 (PMC6003167; doi:10.1186/s12913-018-3240-5)
Supplement: Supplementary file 1 — SSPARS BMC Staff survey. (DOC 28 kb) [file 12913_2018_3240_MOESM1_ESM.doc]

Work location

Team

Discipline:

Role description

**Staff Survey Psychosis and Rehabilitation Stream**

Background

The comprehensive care of people with psychosis involves the use of pharmacotherapy and evidenced based psychosocial therapies (cognitive behavioural therapy for psychosis CBTp, cognitive remediation CRT, Family therapy) and case management.

This survey is to ascertain your current training and interest in training or supporting CBTp and CRT programs in your service.

Thank you for completing this survey

Dr Frances Dark

**Cognitive behaviour therapy for Psychosis CBTp**

1. Have you had training in CBT? Please specify (uni, post graduate, short course, accredited course)

1a. Do you use CBT principles in your current practice?

Please give an example/s

1. Have you had training in CBTp?

Please specify as above

1. Are you currently a CBT therapist (i.e. Seeing people for specific, structured CBT group or individually)
2. Are you currently delivering therapy derived from CBT e.g. DBT, SCIT, ACT?

Please specify

1. DBT
2. SCIT
3. ACT
4. Motivational interviewing
5. CBT group
6. Do you currently receive supervision if conducting CBT/CBTp Y/N : comments

We are interested in training more staff to become CBTp therapists. This would require a co-facilitating a CBT group and seeing at least 2 people for individual therapy in the year following training. Supervision would be provided via peer group supervision (analogous to DBT) and individual supervision as required.

Therapy and supervision time will be quarantined and would be at least 3 hours a week.

1. Considering the above commitment, are you interested in further training to become a CBT therapist? Y/N

Comments

1. Do you have any suggestions or advise or opinions about the implementation & dissemination of CBTp & CRT
2. Have you an interest in any other psychosocial therapies for psychosis?

**Cognitive Remediation**

1. Are you interested in knowing more about the cognitive impairment of psychosis? Y/N
2. Are you interested in learning more about the routine assessment of cognition?

Y/N

1. I am interested in CRT awareness and cognitive compensatory strategy training? Y / N
2. Have you had CRT training?

Specify including date of training

We are interested in developing a “pool” of CRT therapists to ensure it is available to everyone with psychosis. Dr Dark can train facilitators. To be accredited as a CRT facilitator you would be required to facilitate at least one group a week (1.5 hours) for a year and attend group supervision monthly while you are running a group.

1. Considering the above, I would be interested in becoming a CRT facilitator. Y/N

Comments

Thank you for completing this survey. I value any input you can give uon improving the care for people with psychosis and maintaining and retaining a skilled and motivated workforce.

Please feel free to email me: frances_dark@health.qld.gov.au
